# Shifts in Home Health Care Delivery for Patients with Dementia Following 2020 Payment Reform in the U.S

**DOI:** 10.1002/alz70860_104367

**Published:** 2025-12-23

**Authors:** Julia G Burgdorf, Tracy Mroz

**Affiliations:** ^1^ Center for Home Care Policy & Research, New York, NY, USA; ^2^ University of Washington, Seattle, WA, USA

## Abstract

**Background:**

In the US, home health (HH) is a crucial source of care for community‐living Persons with Dementia (PwD). One‐third of HH patients have diagnosed dementia. In 2020, Medicare (federal insurance for older adults) adopted a new HH payment system: the Patient‐Driven Groupings Model (PDGM). PDGM does not include dementia in risk adjustment and prior payment reforms decreased HH access and quality for PwD. Our analysis is the first work to examine PDGM's impact on HH care delivery for PwD.

**Methods:**

Our analytic sample included older adults (65+) who received HH in 2019 or 2021 and were referred to HH from the community (*n* = 907,553). Using linked 2019 & 2021 Medicare HH claims, patient assessment, and provider data, we examined the services patients received as a function of the interaction between dementia diagnosis and year (i.e., before or after PDGM implementation). HH includes six services: nursing, physical therapy, occupational therapy, speech‐language therapy, social work, and aide. We used multivariable logistic regression to model odds of receiving each service. We used multivariable negative binomial regression to model the total number of visits received. All models adjusted for patient sociodemographic, clinical, and functional characteristics, and HH provider characteristics.

**Results:**

Among our sample, 30.9% had diagnosed dementia, mean age was 81.5 years (SD: 8.3), and 71.5% were female. Compared to *before* PDGM implementation, patients with dementia *after* PDGM had lower odds of receiving nursing (Adjusted Odds Ratio (aOR): 0.74; 95% Confidence Interval (CI): 0.72‐0.75), occupational therapy (aOR: 0.81; 95% CI: 0.79‐0.83), speech‐language therapy (aOR: 0.89; 95% CI: 0.87‐0.93), social work (aOR: 0.72; 95% CI: 0.70‐0.75), and aide services (aOR: 0.69; 95% CI: 0.67‐0.71). Patients with dementia *after* PDGM also received fewer total visits compared to *before* PDGM (Adjusted Incidence Rate Ratio: 0.85; 95% CI: 0.85‐0.86).

**Conclusions:**

Following implementation of a new HH payment system (PDGM), patients with dementia are less likely to receive services, including those most relevant to care for PwD such as occupational therapy, speech‐language therapy, and social work. These unintended consequences may threaten HH quality and the ability to safely age in place for the high‐need population of community‐living PwD.

